# Perceived communication quality of a standardized neuroradiology report following AI-based simplification: a randomized, blinded study

**DOI:** 10.1186/s13244-026-02391-5

**Published:** 2026-08-27

**Authors:** Agreen Horr, Isabella Luisa Walther, Alexandra Bonietzki, Tim-Christian Piesch, Sönke Peters, Mehmet Mert Sarikaya, Svea Seehafer, Olav Jansen, Patrick Langguth, Schekeb Aludin

**Affiliations:** 1 Department of Radiology and Neuroradiology, University Medical Center Schleswig-Holstein, Kiel, Germany; 2 Department of Vascular and Endovascular Surgery, University Medical Center Schleswig-Holstein, Kiel, Germany

**Keywords:** Generative artificial intelligence, Large language models, Radiology, Health Literacy, Patient-centered care

## Abstract

**Objectives:**

Radiology reports are primarily written for professional communication and may be difficult for patients to understand. We investigated whether AI-based simplification of a single standardized neuroradiology report improves participant-rated communication quality compared with a conventional professional-language report.

**Materials and methods:**

In this prospective, randomized, blinded study, 100 adults received either an AI-simplified or a conventional report based on the same standardized neuroradiology case. Two board-certified radiologists reviewed the simplified version before evaluation. Participants rated clarity and structure, tone and trust, usability, and overall impression on structured 5-point scales. No objective comprehension test was performed. Group differences were assessed using Welch’s *t*-tests, with effect sizes reported as Cohen’s *d*; Mann–Whitney *U*-tests served as sensitivity analyses. Open-ended responses were analyzed qualitatively.

**Results:**

Participants rated the AI-simplified report more favorably across all domains (all *p* < 0.001). Effect sizes were large to very large, ranging from *d* = 1.31 for Tone and Trust to *d* = 2.10 for Overall Impression. Nonparametric sensitivity analyses confirmed all group differences. Qualitative responses likewise emphasized clearer explanations, simpler language, and improved structure.

**Conclusion:**

In this single standardized neuroradiology case, AI-based simplification improved participant-rated communication quality. Studies using multiple cases and outputs should determine whether such perceived improvements translate into objective comprehension, recall, decision-making, and clinically meaningful communication benefits.

**Key Points:**

- **Question** Can radiologist-reviewed AI simplification address the communication gap by improving perceived communication quality of a standardized neuroradiology report compared with a conventional professional-language report?
- **Findings** In this randomized study with concealed AI involvement, the AI-simplified report received higher ratings across all four participant-rated communication domains.
- **Critical relevance statement** In a standardized neuroradiology report, AI-based simplification improved perceived communication quality, supporting further evaluation of patient-facing report explanations as adjuncts to conventional reports with explicit quality-assurance criteria.

**Graphical Abstract:**

**Figure:**
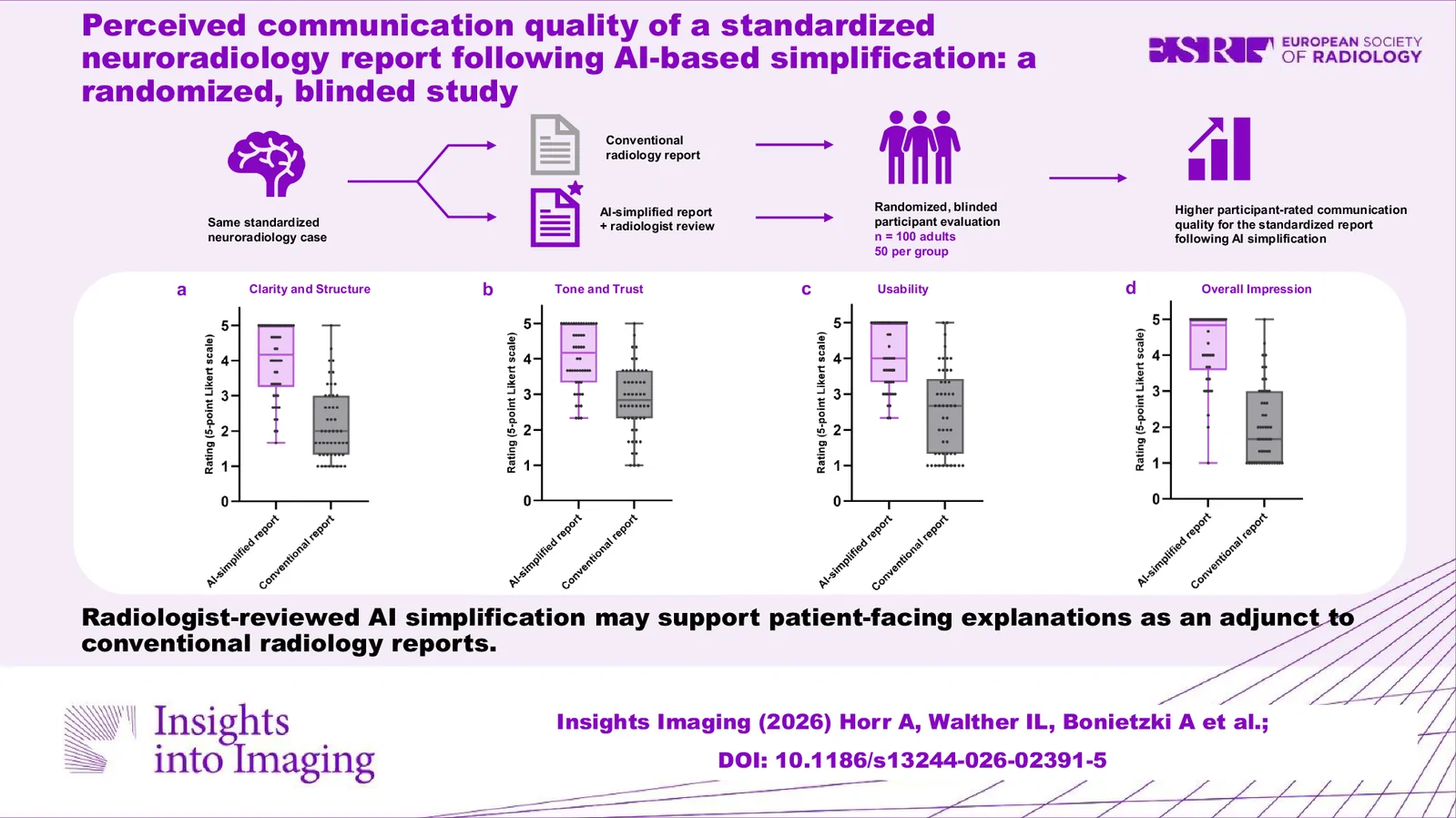

## Introduction

Radiology reports are central to diagnostic care and are primarily written for communication among healthcare professionals [1–4]. With the widespread use of electronic health records and patient portals, patients increasingly access them directly [5, 6]. However, linguistic complexity and frequent use of specialized terminology may limit patient understanding and confidence in interpreting findings [7–9]. Limited comprehensibility may hinder patient-centered communication by contributing to uncertainty, misunderstanding, and reduced engagement in medical decision-making [1, 8, 10, 11].

Large language models (LLMs) can transform technical medical text into more patient-friendly language [3, 12]. Previous studies have shown that LLM-based simplification can improve the readability of radiology reports across imaging contexts, often using established readability metrics as primary outcomes [13, 14]. Emerging evidence also suggests improvements in objective comprehension and perceived clarity, although concerns regarding trust in AI-generated report explanations remain [7, 15].

AI-based report simplification also carries risks. Simplified versions may lose clinically relevant nuance, inadequately convey diagnostic uncertainty, or introduce semantic shifts that could lead to misinterpretation or misinformation [3, 16–20]. Comparative evaluations have shown variability in structure, readability, understandability, and communication features across different LLMs [21]. These considerations support evaluating AI-based simplification as a patient-facing adjunct to conventional radiology reporting with explicit quality-assurance criteria and expert oversight.

Beyond linguistic simplification, implementation-relevant questions remain. It is unclear how readers perceive the communication quality of a radiologist-reviewed AI-simplified report when blinded to the use of AI. This distinction is important because trust-related ratings may otherwise reflect attitudes toward AI rather than the report text itself. Therefore, this randomized, blinded participant study evaluated whether AI-based simplification of a single standardized neuroradiology report improves participant-rated communication quality compared with a conventional professional-language report.

## Materials and methods

### Ethical considerations

The study was conducted in accordance with the Declaration of Helsinki and approved by the local institutional ethics committee (reference number D 492/25). All participants provided informed consent before participation. Participation was voluntary and anonymous, and no directly identifiable data were collected.

### Study design and setting

We conducted a prospective, randomized, controlled parallel-group study at a tertiary academic medical center in Germany. Participants anonymously completed a standardized questionnaire after reading either an AI-simplified or conventional report. Data were collected from June 1 to December 31, 2025, when recruitment ended as planned. Because this was a questionnaire-based participant evaluation, no formal a priori sample size calculation was performed; the sample size was determined pragmatically based on the predefined recruitment period and feasibility of consecutive recruitment. The study was conducted and reported in accordance with the Consolidated Standards of Reporting Trials (CONSORT) recommendations for randomized trials [22]. A completed checklist is provided separately; some items apply only indirectly to this questionnaire-based evaluation of a single standardized case.

### Participants

Participants were recruited consecutively from the radiology waiting area between June 1 and December 31, 2025. Eligible individuals were adults aged 18 years or older who could read and understand German and provided written informed consent. Individuals younger than 18 years or those who declined participation were excluded. Demographic information, including age, sex, highest reported educational level, professional background, and self-reported prior experience with radiology reports, was recorded anonymously.

### Randomization and allocation

**Fig. 1:**
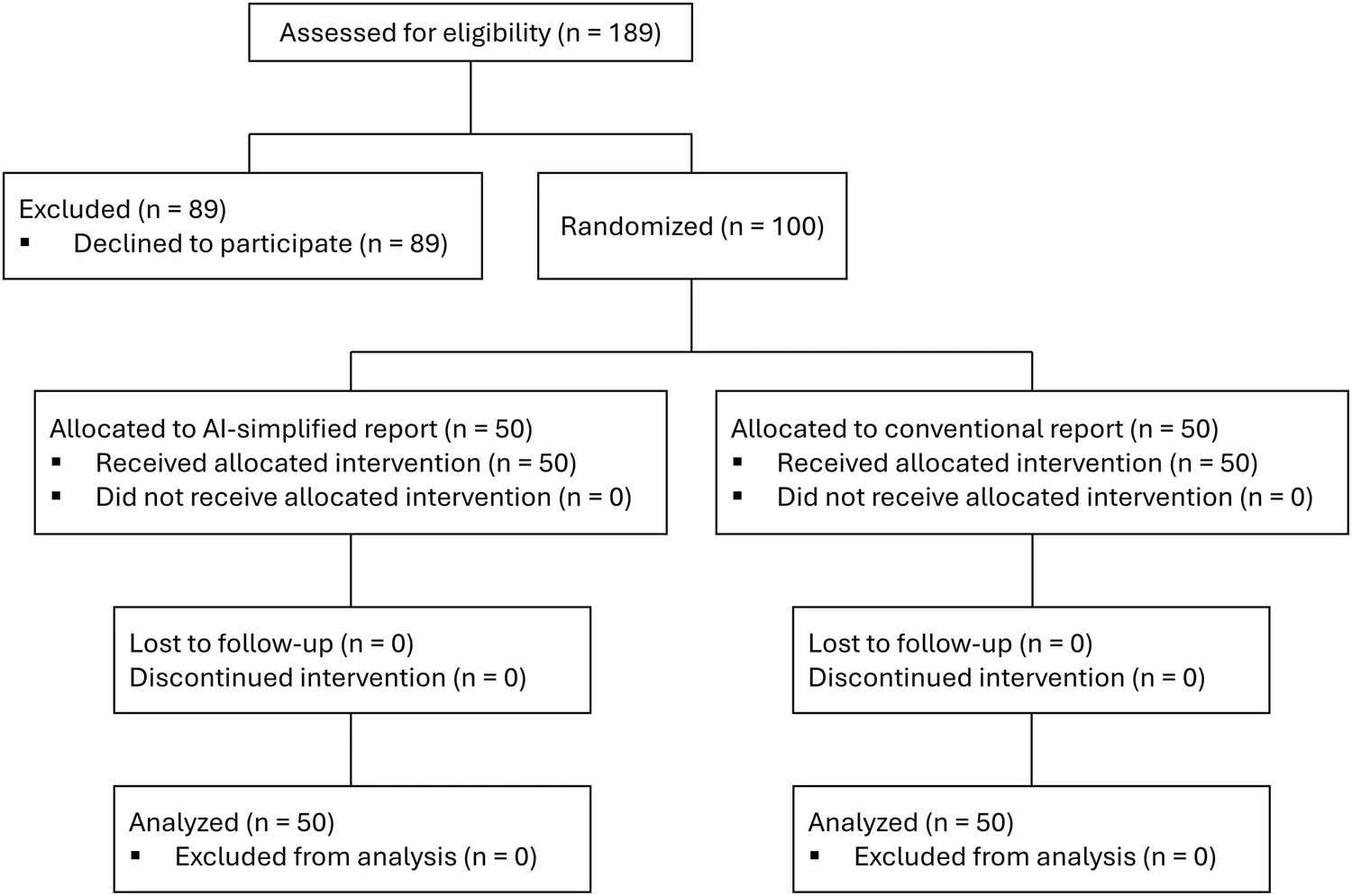
CONSORT flow diagram of participant recruitment, randomization, allocation, follow-up, and analysis. The diagram shows the number of individuals assessed for eligibility, excluded before randomization, allocated to the AI-simplified and conventional report groups, followed up, and included in the final analysis. AI, artificial intelligence

Participants were assigned in a 1:1 ratio to the AI-simplified or conventional report group using randomly intermixed paper questionnaire versions. Before recruitment, equal numbers of both versions were prepared, randomly mixed, and distributed sequentially, with each participant receiving the next available version. No block randomization was used. Investigators distributing the questionnaires were unaware of the upcoming allocation. Participants were blinded to the study hypothesis, group allocation, and the use of AI-based simplification. However, because the report versions intentionally differed in language, structure, and explanatory detail, blinding did not extend to the communication format itself. Each participant evaluated only one report version. Participant flow is shown in Fig. 1.

### Intervention and control conditions

The control condition consisted of a conventional German neuroradiology report written in standard professional medical language. The intervention was a linguistically simplified version of the same report generated using GPT-4o (OpenAI, San Francisco, CA, USA) via a custom GPT within the ChatGPT interface. The custom GPT was guided by a predefined rule-based prompt framework developed by the authors, drawing on established models of comprehensible communication and text simplification [23, 24].

Simplification aimed to preserve the diagnostic content of the conventional report while improving accessibility for non-medical readers through plain-language wording, brief explanations of medical terminology, shorter sentences, and clearer organization. The two report versions therefore differed primarily in linguistic formulation, explanatory detail, and structure. Details on the custom GPT configuration, prompting language, date of use, generation process, radiologist review, post-review edits, and prompt framework are provided in Supplementary Material 1.

Supplementary Material 2 provides the full conventional and AI-simplified reports, including the original German texts used in the participant questionnaire and English translations for reader convenience.

### Radiologist review of the AI-simplified report

Before participant evaluation, the AI-simplified report was reviewed as an internal quality-assurance step by two board-certified radiologists with more than 10 years of clinical experience. The reviewers assessed whether the simplified version preserved the relevant diagnostic content and clinical meaning of the conventional report, whether any relevant findings had been omitted or added, and whether the wording was appropriate for non-medical readers. The reviewed version was subsequently used unchanged in the participant evaluation.

### Characteristics of the radiology case example

The evaluated text was based on a single standardized, fictitious brain MRI report reflecting the structure and complexity of routine neuroradiology reporting. It described an extra-axial mass consistent with a meningioma and incidental findings including an arachnoid cyst, evidence of an old infarction, mild microangiopathic changes, and nonspecific paranasal sinus mucosal thickening.

### Questionnaire and outcomes

A structured questionnaire was used to obtain participant-reported evaluations, including demographic items, quantitative rating scales, and open-ended feedback. The four questionnaire scales were prespecified as exploratory participant-rated outcome domains, and no formal hierarchy of primary and secondary outcomes was defined.

The quantitative assessment comprised four scales with three items each:

- Clarity and structure: overall understanding, comprehensibility of terminology, clarity and logical structure.
- Tone and trust: empathy and respectful language, professionalism and trustworthiness, uncertainty after reading (reverse-coded).
- Usability: layout, ability to identify key information quickly, supportive effect of headings and structure.
- Overall impression: feeling adequately informed, preference for similar reports in the future, and overall usefulness and comprehensibility.

All items were rated on a 5-point Likert scale (1 = strongly disagree, 5 = strongly agree). Open-ended responses were analyzed using qualitative content analysis according to Mayring and subsequently summarized descriptively [25].

### Data handling and statistical analysis

Statistical analyses were performed using jamovi (Version 2.7.15; The jamovi project, Sydney, Australia). Scale scores were calculated as the mean of the respective items. Before analysis, data were screened for completeness and plausibility. Missing item-level data were handled using person-mean imputation within each scale, provided that at least two of three items were available. Internal consistency of the questionnaire scales was assessed using Cronbach’s α. To describe baseline comparability, absolute standardized differences were calculated for baseline characteristics, using standardized mean differences for age and standardized differences in proportions for categorical variables. In line with the exploratory outcome structure, group comparisons were conducted as exploratory analyses of the prespecified participant-rated outcome domains. Differences between participants receiving the AI-simplified report and those receiving the conventional report were analyzed using Welch’s *t*-tests. Effect sizes were reported as Cohen’s *d* with corresponding 95% confidence intervals (CI). Because Likert-type ratings may not fully satisfy assumptions of interval-scaled data, Mann–Whitney *U*-tests were performed as nonparametric sensitivity analyses to assess the robustness of the group comparisons. Additional post hoc exploratory effect-modification analyses were performed using linear regression models including report group, the potential effect modifier, and the corresponding group-by-modifier interaction term. Potential effect modifiers were age, entered as a continuous variable in years, university degree vs no university degree, and medical professional background. All statistical tests were two-sided, and a significance level of α = 0.05 was applied. Graphical representations were created using GraphPad Prism (Version 10.6.1; GraphPad Software, Boston, MA, USA).

## Results

### Participant flow

A total of 189 individuals were assessed for eligibility. Of these, 89 declined to participate. The remaining 100 participants were randomized and allocated in a 1:1 ratio to the AI-simplified report group (*n* = 50) or the conventional report group (*n* = 50). All participants received the allocated report version. No participants were lost to follow-up or discontinued the study. All randomized participants were analyzed in the groups to which they were assigned (Fig. 1).

### Participant characteristics

Baseline demographic characteristics of the analyzed cohort are shown in Table 1, including absolute standardized differences. The mean age of the cohort was 64.4 ± 16.2 years. The sample comprised 38 women, 60 men, and 2 non-binary participants. Most participants had a non-medical professional background (89%), and approximately half reported prior experience with radiology reports. Age and medical professional background were well balanced between groups, whereas differences were observed for sex, prior experience with radiology reports, and individual educational categories.

**Table 1:** Demographic and baseline characteristics of the study population

Characteristic	Total (*N* = 100)	AI group (*n* = 50)	Conventional group (*n* = 50)	Std. diff.
Age (years)				
Mean ± SD	64.38 ± 16.22	64.34 ± 18.58	64.42 ± 13.66	0.00
Range	21–94	21–94	28–92	—
Sex				
Female	38 (38)	22 (44)	16 (32)	0.25
Male	60 (60)	27 (54)	33 (66)	0.25
Non-binary	2 (2)	1 (2)	1 (2)	0.00
Highest educational level				
No school certificate	2 (2)	2 (4)	0 (0)	0.29
Secondary school	28 (28)	13 (26)	15 (30)	0.09
High school diploma	10 (10)	4 (8)	6 (12)	0.13
Vocational training	38 (38)	21 (42)	17 (34)	0.17
University degree	22 (22)	10 (20)	12 (24)	0.10
Medical background				
Medical profession	11 (11)	5 (10)	6 (12)	0.06
Non-medical profession	89 (89)	45 (90)	44 (88)	0.06
Previous experience with radiology reports				
None	47 (47)	26 (52)	21 (42)	0.20
Once	24 (24)	9 (18)	15 (30)	0.28
Multiple times	29 (29)	15 (30)	14 (28)	0.04

Data are presented as *n* (%) unless otherwise indicated. Percentages are calculated within columns. In case of multiple responses for educational level, the highest reported level was used for categorization. Absolute standardized differences are reported as standardized mean differences for age and standardized differences in proportions for categorical variables

*AI* artificial intelligence, *SD* standard deviation, *Std. diff.* absolute standardized difference

### Internal consistency

All four questionnaire scales (Clarity and Structure, Tone and Trust, Usability, and Overall Impression) demonstrated acceptable to excellent internal consistency according to commonly used thresholds [26]. Cronbach’s α ranged from 0.771 to 0.970 (Table 2).

**Table 2:** Internal consistency of questionnaire scales

Scale	Number of items	Cronbach’s α
Clarity and structure	3	0.939
Tone and trust	3	0.771
Usability	3	0.926
Overall impression	3	0.970

Cronbach’s α was used to assess internal consistency. Values ≥ 0.70 were considered acceptable, ≥ 0.80 good, and ≥ 0.90 excellent [26].

### Quantitative outcomes

**Fig. 2:**
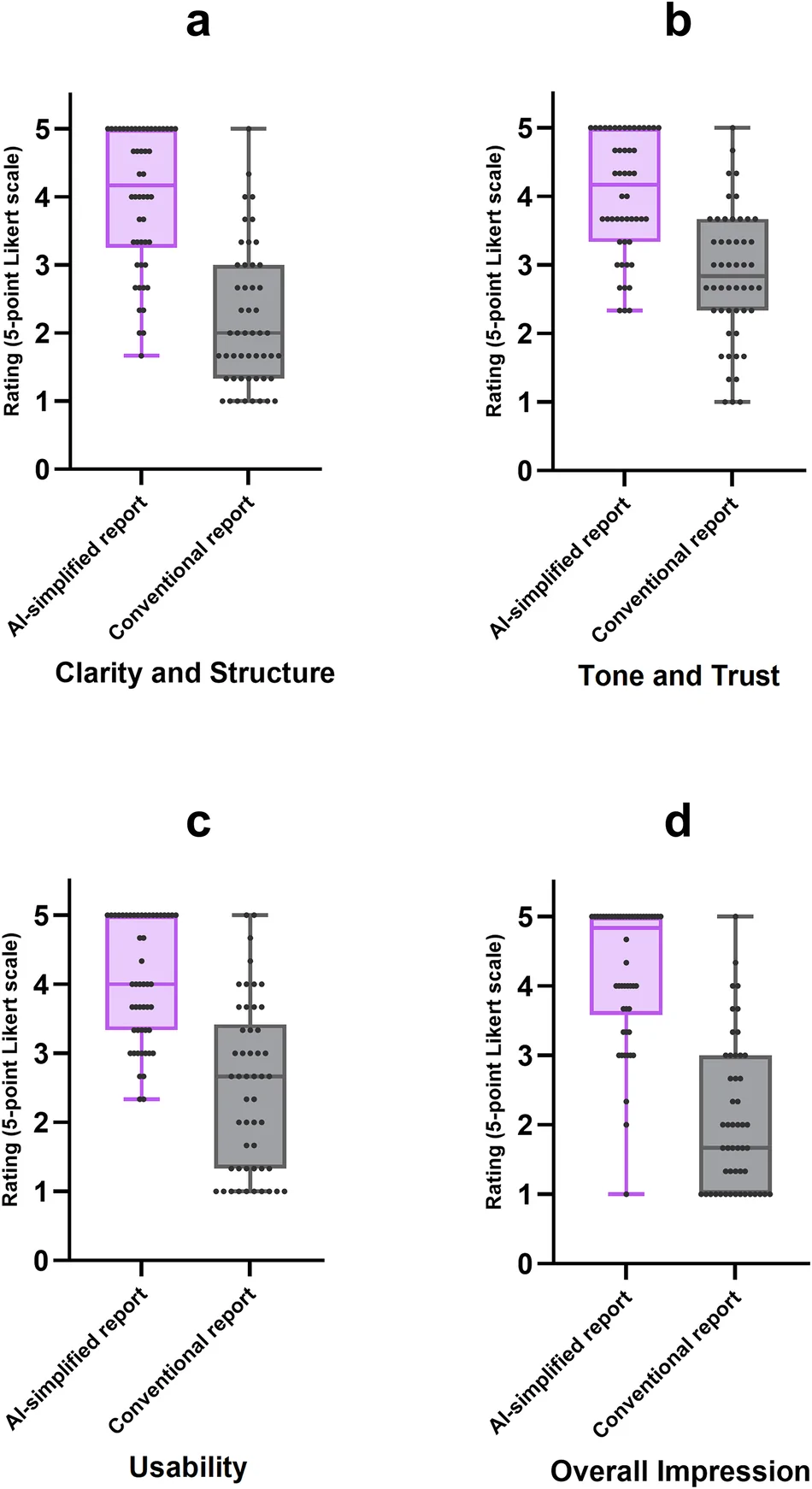
Participant-rated communication outcomes. Boxplots with overlaid individual participant scale scores comparing the AI-simplified and conventional radiology reports for **a** clarity and structure, **b** tone and trust, **c** usability, and **d** overall impression. Scale scores were calculated as the mean of three items rated on a 5-point Likert scale, with higher values indicating more favorable evaluations. The central line represents the median, boxes indicate the interquartile range (IQR), whiskers indicate the minimum and maximum values, and dots represent individual participant scale scores. Purple boxes represent the AI-simplified report and gray boxes represent the conventional report. Each group included 50 participants. AI, artificial intelligence

Across all evaluated dimensions, participants who received the AI-simplified report rated the text significantly more positively than those who read the conventional report (Table 3 and Fig. 2). Mean scores were consistently higher in the AI-simplified group for clarity and structure (4.00 ± 1.03 vs 2.17 ± 1.01), tone and trust (4.03 ± 0.88 vs 2.83 ± 0.96), usability (4.05 ± 0.89 vs 2.49 ± 1.21), and overall impression (4.21 ± 0.98 vs 2.05 ± 1.07).

**Table 3:** Descriptive statistics of questionnaire scales by study group

Scale	Group	*N*	Mean	Median	SD
Clarity and structure	AI-simplified report	50	4.00	4.17	1.03
	Conventional report	50	2.17	2.00	1.01
Tone and trust	AI-simplified report	50	4.03	4.17	0.88
	Conventional report	50	2.83	2.83	0.96
Usability	AI-simplified report	50	4.05	4.00	0.89
	Conventional report	50	2.49	2.67	1.21
Overall impression	AI-simplified report	50	4.21	4.83	0.98
	Conventional report	50	2.05	1.67	1.07

Values are reported as mean, median, and standard deviation (SD). Ratings were assessed on a 5-point Likert scale, with higher values indicating more favorable ratings

*AI* artificial intelligence, *SD* standard deviation

Welch’s *t*-tests confirmed statistically significant group differences for all outcome measures (all *p* < 0.001; Table 4). Effect sizes were large to very large across all scales. For Clarity and Structure, the AI-simplified group showed a very large effect (*d* = 1.79, 95% CI: 1.32–2.25). Similarly, large effects were observed for Tone and Trust (*d* = 1.31, 95% CI: 0.87–1.74) and Usability (*d* = 1.46, 95% CI: 1.02–1.90). The strongest effect was found for Overall Impression (*d* = 2.10, 95% CI: 1.61–2.59).

**Table 4:** Group comparisons between the AI-simplified and conventional report groups

Scale	Welch’s t (d*f*)	*p* value	Cohen’s *d* (95% CI)
Clarity and structure	8.95 (98.0)	< 0.001	1.79 (1.32–2.25)
Tone and trust	6.53 (97.3)	< 0.001	1.31 (0.87–1.74)
Usability	7.31 (90.1)	< 0.001	1.46 (1.02–1.90)
Overall impression	10.52 (97.3)	< 0.001	2.10 (1.61–2.59)

Values represent Welch’s *t*-tests for independent samples comparing the AI-simplified and conventional report groups. Effect sizes are reported as Cohen’s *d* with 95% CIs. Positive effect sizes indicate higher ratings in the AI-simplified group

*AI* artificial intelligence, *CI* confidence interval, *df* degrees of freedom

Missing item-level responses were rare. Overall, 14 of 1200 item-level responses were missing (1.17%), including 5 in the AI-simplified group and 9 in the conventional-report group. No scale-level values were missing after person-mean imputation, and all scale-level analyses included 50 participants per group. Nonparametric Mann–Whitney *U* sensitivity analyses confirmed the direction and statistical significance of the group differences; median and interquartile range values together with U statistics are provided in Supplementary Table 1.

Post hoc exploratory effect-modification analyses did not show evidence that the effect of report group differed by age, university degree, or medical professional background. Across the four participant-rated outcome domains, all group-by-modifier interaction terms were non-significant, with *p* values ≥ 0.204 for age, ≥ 0.275 for university degree, and ≥ 0.307 for medical professional background (Supplementary Table 2).

### Qualitative findings

Open-ended responses supported the quantitative findings. Participants in the AI-simplified group frequently emphasized clearer explanations, simpler language, and improved structure. In contrast, participants who read the conventional report often described the text as difficult to understand because of technical terminology and reported that additional explanations or independent information seeking would have been necessary. Representative examples and the coding framework are provided in Table 5.

**Table 5:** Categories and coding rules for open-ended feedback

Free-text question	Category	Coding rule	Representative example
Helpful aspects of the report	Explanations	Statements referring to the helpfulness of explanations.	*“The simple explanations were supportive”.*
	Clear language	Statements indicating facilitated understanding due to clear language.	*“The language is easy to understand”.*
	Previous knowledge	References to prior knowledge as beneficial for understanding.	*“I only understood the report due to my prior knowledge”.*
Suggestions for improvement	No suggestions for improvement	Statements expressing no need for improvement or highlighting only positive aspects.	*“Everything is perfect, keep going!”*
	Language complexity	Statements referring to difficulties caused by technical wording or terminology.	*“Too many medical expressions”.*
	Need for further explanations/glossary	Statements indicating the need for additional explanations or a glossary.	*“More explanations.”/“Please add a glossary for technical terms”.*
Additional comments/future preference	Need for external help	References to the need for additional or external support.	*“To understand this report, I would have needed to Google nearly everything”.*
	Wish for similar reports in the future	Expressions of preference for similar reports in the future.	*“More medical reports should be written like this”.*

Categories were derived from qualitative content analysis according to Mayring [25]. Representative examples are shown. Representative quotations were translated from German into English by the authors.

## Discussion

### Principal findings

This randomized, blinded participant study showed that AI-based linguistic simplification of a single standardized neuroradiology report significantly improved participant-rated communication quality across Clarity and Structure, Tone and Trust, Usability, and Overall Impression. Because the conventional report was written for professional communication whereas the simplified version was designed as a patient-facing explanation, the large effects should be interpreted in light of the deliberately different target audiences, explanatory detail, structure, and language of the two report versions.

Clear and understandable medical information is important for effective doctor-patient communication [27, 28]. As patients increasingly access radiology reports directly through electronic health records and patient portals, the linguistic gap between professional reporting and patient understanding becomes more relevant [5, 6]. In this context, AI-assisted report simplification may help patients perceive radiological information as more understandable and may support patient-centered communication before or during clinical consultations, without replacing professional interpretation [7, 15, 29].

### Comparison with prior work

This randomized, blinded participant study extends previous work by evaluating participant-rated communication quality under concealed AI involvement. Prior studies have shown that LLMs can improve readability, comprehensibility, objective understanding, and patient-reported perceptions of radiology reports [3, 7, 13–15, 29]. Concealment of AI involvement is particularly relevant for Tone and Trust. While a previous study reported lower trust in AI-translated report explanations [15], participants in the present study were blinded to the use of AI. Accordingly, the higher Tone and Trust ratings observed for the AI-simplified version should be interpreted primarily as responses to the simplified text rather than as responses to an explicit AI label. However, these ratings require cautious interpretation. Higher trust-related ratings for a simpler and more fluently processed text do not necessarily indicate warranted trust or greater diagnostic reliability. They may partly reflect processing fluency, perceived clarity, and the more accessible organization of the patient-facing explanation.

The consistently large to very large effects were supported by qualitative feedback indicating that participants perceived the AI-simplified report as clearer, more accessible, and more useful than the conventional version. These advantages likely reflect the combined effects of plain-language reformulation, explanatory additions, shorter sentence structures, and improved organization rather than terminology simplification alone.

The magnitude of the observed effects may partly reflect the high linguistic complexity of conventional radiology reports, which are primarily designed for professional communication rather than for lay readers [1–4, 9]. This challenge has also been described as the “curse of knowledge” in radiology reporting, whereby expert-oriented language may unintentionally hinder understanding among patients and referring physicians [30]. Such reports may therefore present a substantial initial barrier for readers without medical training, potentially contributing to the differences between the two report versions [1, 4, 7, 15]. The structured layout and explanatory passages included in the AI-simplified version may have further improved usability and perceived clarity, consistent with the Patient Education Materials Assessment Tool (PEMAT), which identifies organization, plain language, and explanations of medical terms as contributors to understandability [31].

### Limitations

Several limitations should be considered. First, the study evaluated a single standardized fictitious neuroradiology report rather than authentic reports from routine practice. This enabled a controlled comparison based on the same underlying case but limits generalizability to other imaging modalities, report types, and clinical scenarios. Because only one conventional report and one AI-simplified counterpart were assessed, the observed effects may also reflect characteristics of this specific report pair. Studies using multiple cases, report types, and independently generated AI outputs are therefore needed before broader conclusions about AI-based simplification can be drawn. The standardized comparison may also have increased the contrast between versions, contributing to floor effects in the control group. In the AI-simplified group, Overall Impression ratings clustered near the upper limit of the 5-point scale, suggesting a ceiling effect that may have reduced discrimination among highly favorable evaluations. Participants were blinded to the study hypothesis, group allocation, and AI involvement, but not to the visible communication format. The findings should therefore be interpreted as responses to the evaluated texts rather than as effects independent of their differing language, structure, and explanatory detail.

Second, perceived understanding and communication quality were assessed using self-reported measures rather than objective knowledge-based tests. No comprehension check was performed; therefore, the findings do not demonstrate improved objective understanding, recall, or clinical decision-making. They instead indicate improved perceived communication quality, which should be complemented by objective comprehension measures in future studies [32].

Third, the questionnaire scales were study-specific and not externally validated, although internal consistency was acceptable to excellent. Recruitment at a single tertiary care center further limits generalizability. Despite randomization, baseline differences were present for sex, prior experience with radiology reports, and individual educational categories. These characteristics may have influenced participant-rated outcomes, and residual confounding cannot be excluded. In addition, 89 of 189 eligible individuals declined participation. Because no information was collected from non-participants, participation bias cannot be excluded. Those who agreed to participate may have differed in their willingness, time, or ability to engage with written medical information, and the sample may therefore not fully represent patients accessing radiology reports in routine care.

Finally, despite radiologist review, the simplified text contained several interpretive additions or semantic shifts. Specifically, it attributed the presenting headache to the meningioma, described cerebral blood supply as normal based on unremarkable intracranial angiography, and interpreted maxillary sinus mucosal thickening as mild chronic sinus inflammation, although these conclusions were not explicitly established in the conventional report. Radiologist review may therefore reduce, but not eliminate, semantic shifts. Future quality-assurance workflows should include explicit criteria for semantic fidelity, preservation of diagnostic uncertainty, and avoidance of unsupported causal attribution before patient-facing use.

### Practical implications

AI-based patient-facing simplification should be evaluated as an adjunct to, rather than a replacement for, conventional radiology reporting. Simplified explanations could be delivered through secure patient portals or institutionally hosted applications [5, 6], provided that data protection, clinical governance, and professional oversight are ensured [17, 18]. The conventional report should remain the authoritative diagnostic document. Because AI-generated simplifications may introduce semantic shifts or omit clinically relevant nuance [3, 16–20], implementation requires structured quality assurance, expert review, constrained prompts, and task-specific validation.

Future studies should use authentic reports across multiple modalities, objective comprehension tests, and real-world clinical workflows. Comparisons with radiologist-authored lay summaries are needed to determine whether AI provides incremental benefit and whether improved perceived communication translates into objective understanding, better consultation preparation, and clinically meaningful patient benefits.

## Conclusions

AI-based simplification of a single standardized neuroradiology report improved participant-rated communication quality. Future studies using multiple cases and outputs should determine whether these perceived benefits translate into objective comprehension and clinically meaningful communication outcomes in real-world settings.

## Supplementary information


ELECTRONIC SUPPLEMENTARY MATERIAL


## Data Availability

The datasets generated and analyzed during the current study are not publicly available due to privacy and ethical restrictions, but anonymized data may be available from the corresponding author upon reasonable request and subject to applicable institutional and ethical requirements.

## Abbreviations

AI: Artificial intelligence
CI: Confidence interval
CONSORT: Consolidated standards of reporting trials
PEMAT: Patient education materials assessment tool
