# Supplementary material for: Perceived communication quality of a standardized neuroradiology report following AI-based simplification: a randomized, blinded study
Source: Insights Imaging. 2026 Aug 27;17:213. doi: 10.1186/s13244-026-02391-5 (PMC13522326; doi:10.1186/s13244-026-02391-5)
Supplement: Supplementary file 1 [file 13244_2026_2391_MOESM1_ESM.pdf]

Perceived communication quality of a standardized neuroradiology report following AI-based simplification: a randomized, blinded study

ELECTRONIC SUPPLEMENTARY MATERIAL

Insights Imaging (2026) Horr A, Walther IL, Bonietzki A et al.

Supplementary Material 1

AI simplification workflow and prompt framework

Model and language
The AI-simplified report was generated using GPT-4o (OpenAI, San Francisco, CA, USA) via a custom-configured GPT within the ChatGPT interface. The prompt and output language were German.

Date of use and model settings
The AI simplification was performed in April 2025 using GPT-4o via a custom GPT within the ChatGPT interface. The specific model snapshot and generation parameters, including temperature, top-p, and seed, were not available in the interface and therefore could not be recorded.

Generation process
The predefined prompt framework was implemented as the custom GPT's instruction set. The conventional report was then entered as the input text, and the AI-simplified report was generated in a single prompting step. No iterative prompting or additional regeneration steps were used before radiologist review.

Radiologist review
The AI-simplified report was subsequently reviewed by two board-certified radiologists with more than 10 years of clinical experience. The review focused on medical accuracy and completeness, preservation of the diagnostic meaning of the conventional report, absence of relevant omitted or added findings, and appropriateness of the wording for non-medical readers.

Post-review edits
After radiologist review, no further changes were made to the AI-simplified report. The reviewed version was used unchanged for participant evaluation.

Prompt framework used for AI-based simplification

The custom GPT was configured to simplify German radiology reports for non-medical readers while preserving the diagnostic content and clinical meaning of the original report. The predefined prompt framework is provided below as an English translation of the German instructions used to configure the custom GPT for report simplification.

Role and task:
You are a language assistant for patient-oriented explanation of radiology reports. Your task is to transform a conventional German radiology report into a clear, understandable German version for non-medical readers.

Input:
A conventional German radiology report.

Output:
A patient-oriented German version of the same report.

Instructions:

1. Preserve all diagnostic findings, anatomical locations, measurements, and diagnostic conclusions from the original report.
2. Do not add new findings, new diagnoses, prognostic statements, or treatment recommendations.
3. Do not omit clinically relevant information.
4. Replace specialized medical terminology with plain-language wording where possible.
5. If a medical term is important, keep the term and add a short explanation in parentheses.
6. Restructure long or complex sentences into shorter and grammatically simpler sentences.
7. Organize the report using clear headings, bullet points, and a short summary.
8. Explain the clinical meaning of relevant findings in cautious, non-alarming language.
9. Clearly distinguish between the main finding and incidental or additional findings.
10. Preserve uncertainty and avoid overstating findings.
11. The simplified version must remain medically faithful to the original report.
12. Use a respectful, neutral, and patient-oriented tone.

Formatting:
Structure the output using clear sections, bullet points, explanatory notes, and a short summary. The simplified report should include the reason for examination, the findings and their meaning, and a concise final summary.

Supplementary Material 2

Full conventional radiology report and corresponding AI-simplified version

Original German report texts used in the participant questionnaire

Conventional radiology report - original German version

Befund
MRT Schädel (cMRT) vom 15.04.2025

Fragestellung
Abklärung neu aufgetretener, persistierender linksseitiger Kopfschmerzen.
Ausschluss einer intrakraniellen Raumforderung.

Voruntersuchung
Es liegen keine vergleichbaren Voraufnahmen vor.

Links parasagittal frontoparietal zeigt sich in der T1w-Postkontrast-Sequenz eine etwa 2,8 × 2,3 × 2,5 cm große, extraaxiale Raumforderung mit homogener Kontrastmittelaufnahme sowie breiter duraler Anlagerung. In den T2w- und FLAIR-Sequenzen zeigt sich ein gering ausgeprägtes perifokales Ödem. Kein Hinweis auf Infiltration des angrenzenden Hirnparenchyms. Keine Mittellinienverlagerung. In der rechten temporalen Region Darstellung einer glatt begrenzten, rundlichen, extraaxialen Formation mit signalisointensivem Verhalten zu Liquor in T1w- und T2w-Sequenzen. Keine Kontrastmittelaufnahme, kein raumforderndes Verhalten. Subkortikal in der linken Insula Nachweis einer kavitätenbildenden Läsion mit gliofibrotischem Randsaum in den T1w- und FLAIR-Sequenzen. Keine diffusionseingeschränkten Areale in der DWI- und ADC-Sequenz. In der FLAIR-Sequenz Darstellung mehrerer punktförmiger Signalalterationen periventrikulär sowie subkortikal beidseits im Marklager. In der T2w-Sequenz beidseits mukosale Verdickung der Kieferhöhlenschleimhäute bis maximal 4 mm, ohne Nachweis von Flüssigkeitsspiegeln oder ossären Destruktionen. Die intrakraniellen Gefäße erscheinen in der TOF-MR-Angiographie unauffällig, kein Nachweis hämodynamisch relevanter Stenosen, Aneurysmen oder vaskulärer Malformationen.

- Extraaxiale Raumforderung links parasagittal frontoparietal mit homogener KM-Aufnahme und duraler Anlagerung, vereinbar mit einem Meningeom. Geringes perifokales Ödem.
- Glatt begrenzte, liquorisointensive Formation rechts temporal, ohne KM-Aufnahme oder raumforderndes Verhalten, vereinbar mit einer Arachnoidalzyste.
- Chronische, kavitätenbildende Läsion der linken Insula mit gliofibrotischem Randsaum, vereinbar mit einem alten Infarkt.
- Punktförmige Marklagerveränderungen periventrikulär und subkortikal, vereinbar mit geringer Mikroangiopathie.
- Mukosale Verdickung der Kieferhöhlenschleimhäute beidseits, kein Anhalt für akute Sinusitis.

AI-simplified report - original German version

Befund
MRT Schädel (cMRT) vom 15.04.2025

Untersuchungsgrund
Die Patientin oder der Patient hat seit Kurzem anhaltende Kopfschmerzen auf der linken Seite. Ziel der Untersuchung war, eine mögliche Ursache im Gehirn - zum Beispiel einen Tumor - auszuschließen.

Befunde und ihre Bedeutung:

1. **Raumforderung (= ungewöhnliches Gewebe) auf der linken Seite, im Bereich zwischen Stirn und Scheitel (parasagittal frontoparietal):**

   - Größe: ca. 2,8 × 2,3 × 2,5 cm.
   - Sie liegt **außerhalb des Gehirngewebes**, also drückt von außen dagegen.
   - Sie nimmt das Kontrastmittel im MRT gut auf, was typisch für ein **Meningeom** ist.

     - **Ein Meningeom** ist ein meist gutartiger (nicht-krebsartiger) Tumor, der von den Hirnhäuten ausgeht.

   - Es ist **ein kleines umgebendes Hirnödem** (Schwellung des Gewebes) vorhanden.
   - Es gibt **keinen Hinweis**, dass der Tumor ins Gehirn eingewachsen ist.
   - Die Mitte des Gehirns ist **nicht verschoben**, was gut ist.

2. **Rechts in der Schläfenregion (temporal) wurde eine rundliche Struktur entdeckt:**

   - Diese hat ähnliche Eigenschaften wie **Gehirnwasser (Liquor).**
   - Sie nimmt **kein Kontrastmittel** auf und verdrängt das umliegende Gewebe nicht.

     - Das spricht für eine **Arachnoidalzyste**, **eine gutartige, flüssigkeitsgefüllte Blase** im Bereich der Hirnhäute.

3. **Im linken Inselbereich (Insula) wurde eine alte Hirnveränderung gefunden:**

   - Es handelt sich um eine **kavitätenbildende Läsion**, also eine Stelle mit einer kleinen Höhle im Gewebe.
   - Sie hat einen **Rand aus Narbengewebe** (gliofibrotischer Randsaum).

     - Das ist typisch für einen **alten Schlaganfall (Infarkt).**

   - Es gibt **keine Anzeichen** für eine neue Schädigung (wie z. B. frischer Sauerstoffmangel).

4. **Im Marklager beidseits (das ist die tiefere Schicht des Gehirns) wurden mehrere kleine Punkte gefunden:**

    - Diese Veränderungen liegen sowohl nah an den Hirnkammern (periventrikulär) als auch weiter außen (subkortikal).

        - Das spricht für eine **leichte Mikroangiopathie** - also **kleine Durchblutungsstörungen in den kleinsten Gefäßen des Gehirns.**
        - Solche Veränderungen sind im **Alter oder bei Bluthochdruck** nicht ungewöhnlich.

5. **Die Kieferhöhlen zeigen Schleimhautverdickungen:**

    - Diese Schleimhaut ist bis zu 4 mm dick, aber es **gibt keine Flüssigkeitsansammlung** und **keine Knochenschäden.**

        - Das spricht für eine **leichte chronische Entzündung** der Nasennebenhöhlen (Sinusitis).

6. **Die Blutgefäße im Gehirn sind unauffällig:**

    - Es wurden keine Verengungen, Aussackungen (Aneurysmen) oder Fehlbildungen der Gefäße gefunden.

        - Das bedeutet: Die Blutversorgung des Gehirns ist normal.

Zusammenfassung:

- Es wurde ein gutartiger Tumor (Meningeom) außerhalb des Gehirns auf der linken Seite gefunden, der vermutlich die Kopfschmerzen verursacht.
- Zusätzlich wurde eine harmlose Flüssigkeitszyste (Arachnoidalzyste) auf der rechten Seite entdeckt.
- Es gibt eine alte Narbe im Gehirn, wahrscheinlich durch einen früheren kleinen Schlaganfall.
- Kleine Gefäßveränderungen deuten auf altersbedingte oder durchblutungsbedingte Veränderungen hin.
- Eine leichte Entzündung der Kieferhöhlen wurde ebenfalls festgestellt.
- Die Blutgefäße im Gehirn sehen normal aus.

Conventional radiology report - English translation

Report
Brain MRI (cMRI) from 15 April 2025

Clinical question
Evaluation of newly developed, persistent left-sided headaches. Exclusion of an intracranial mass.

Previous examination
No comparable prior imaging is available.

In the left parasagittal frontoparietal region, the T1-weighted post-contrast sequence shows an approximately 2.8 × 2.3 × 2.5 cm extra-axial mass with homogeneous contrast enhancement and broad dural attachment. The T2-weighted and FLAIR sequences show mild perifocal edema. No evidence of infiltration of the adjacent brain parenchyma. No midline shift. In the right temporal region, there is a smoothly marginated, rounded extra-axial formation with signal intensity similar to cerebrospinal fluid on T1-weighted and T2-weighted sequences. No contrast enhancement and no mass effect. Subcortically in the left insula, there is a cavitary lesion with a gliofibrotic rim on T1-weighted and FLAIR sequences. No areas of restricted diffusion on DWI and ADC sequences. The FLAIR sequence shows several punctate signal alterations in the periventricular and bilateral subcortical white matter. The T2-weighted sequence shows bilateral mucosal thickening of the maxillary sinus mucosa up to a maximum of 4 mm, without evidence of fluid levels or osseous destruction. The intracranial vessels appear unremarkable on TOF MR angiography, with no evidence of hemodynamically relevant stenosis, aneurysm, or vascular malformation.

- Extra-axial mass in the left parasagittal frontoparietal region with homogeneous contrast enhancement and dural attachment, consistent with a meningioma. Mild perifocal edema.
- Smoothly marginated, CSF-isointense formation in the right temporal region, without contrast enhancement or mass effect, consistent with an arachnoid cyst.
- Chronic cavitary lesion of the left insula with a gliofibrotic rim, consistent with an old infarction.
- Punctate periventricular and subcortical white matter changes, consistent with mild microangiopathy.
- Bilateral mucosal thickening of the maxillary sinus mucosa, with no evidence of acute sinusitis.

AI-simplified report – English translation

Report
Brain MRI (cMRI) from 15 April 2025

Reason for examination
The patient has recently developed persistent headaches on the left side. The aim of the examination was to rule out a possible cause in the brain - for example, a tumor.

Findings and their meaning:

1. **Mass (= unusual tissue) on the left side, in the region between the frontal and parietal areas (parasagittal frontoparietal):**

   - Size: approx. 2.8 × 2.3 × 2.5 cm
   - It is located **outside the brain tissue,** meaning that it presses against the brain from the outside.
   - It takes up contrast agent well on MRI, which is typical of a **meningioma**.

     - ➢ **A meningioma** is a usually benign (non-cancerous) tumor that arises from the meninges.

   - There is **a small amount of surrounding brain edema** (swelling of the tissue).
   - There is **no evidence** that the tumor has grown into the brain.
   - The midline of the brain is **not shifted**, which is a good sign.

2. **A rounded structure was found on the right side in the temporal region:**

   - It has properties similar to **cerebrospinal fluid.**
   - It does **not take up contrast agent** and does not displace the surrounding tissue.

     - ➢ This suggests an **arachnoid cyst, a benign fluid-filled cyst** in the area of the meninges.

3. **An old brain change was found in the left insular region:**

   - This is a **cavitary lesion,** meaning an area with a small cavity in the tissue.
   - It has a **rim of scar tissue** (gliofibrotic rim).

     - ➢ This is typical of an **old stroke (infarction).**

   - There are **no signs** of new injury (such as recent oxygen deprivation).

4. **Several small spots were found in the white matter on both sides (this is the deeper layer of the brain):**

- These changes are located both close to the brain ventricles (periventricular) and farther outward (subcortical).

    - This suggests **mild microangiopathy** - meaning **small circulatory disturbances in the smallest blood vessels of the brain.**
    - Such changes are not unusual with **increasing age or high blood pressure.**

5. **The maxillary sinuses show mucosal thickening:**

    - The mucosa is up to 4 mm thick, but there is **no fluid accumulation** and **no bone damage.**

        - This suggests **mild chronic inflammation** of the paranasal sinuses (sinusitis).

6. **The blood vessels in the brain are unremarkable:**

    - No narrowing, outpouchings (aneurysms), or vascular malformations were found.

        - This means that the blood supply to the brain is normal.

Summary:

- A benign tumor (meningioma) was found outside the brain on the left side and is probably causing the headaches.
- In addition, a harmless fluid-filled cyst (arachnoid cyst) was found on the right side.
- There is an old scar in the brain, probably from a previous small stroke.
- Small vessel changes suggest age-related or circulation-related changes.
- Mild inflammation of the maxillary sinuses was also found.
- The blood vessels in the brain look normal.

Supplementary Table 1

Scale	Missing responses, AI-simplified	Missing responses, conventional	AI-simplified median [IQR]	Conventional median [IQR]	Mann–Whitney U	p value
**Clarity and Structure**	0	3	4.17 [1.67]	2.00 [1.58]	289	< 0.001
**Tone and Trust**	2	2	4.17 [1.58]	2.83 [1.25]	466	< 0.001
**Usability**	1	1	4.00 [1.67]	2.67 [2.00]	414	< 0.001
**Overall Impression**	2	3	4.83 [1.33]	1.67 [1.92]	219	< 0.001
**Total**	**5**	**9**	—	—	—	—

Supplementary Table 1.
Missing item-level responses and nonparametric sensitivity analyses.
Missing values refer to item-level responses before person-mean imputation. No scale-level values were missing after imputation. Median and interquartile range (IQR) values are reported for each scale and group. Mann–Whitney U statistics and p values refer to comparisons between the AI-simplified report group and the conventional report group. Ratings were assessed on a 5-point Likert scale, with higher values indicating more favorable ratings.
AI = artificial intelligence; IQR = interquartile range

Supplementary Table 2

Outcome domain	Age (continuous), p value	University degree, p value	Medical professional background, p value
**Clarity and Structure**	0.204	0.633	0.379
**Tone and Trust**	0.612	0.981	0.395
**Usability**	0.911	0.902	0.307
**Overall Impression**	0.850	0.275	0.353

Supplementary Table 2.
Post hoc exploratory effect-modification analyses
P values refer to group-by-modifier interaction terms from post hoc exploratory linear regression models including report group (AI-simplified versus conventional report), the respective potential effect modifier, and the corresponding interaction term. University degree was analyzed as university degree versus no university degree. Medical professional background was analyzed as medical profession versus non-medical profession.
